# A novel needle insertion technique for electromyography targeting the flexor digitorum profundus (Digits 2 and 3): A cadaveric validation study

**DOI:** 10.1097/MD.0000000000051278

**Published:** 2026-10-30

**Authors:** Hong Bum Park, Se Ryeon Lee, Jun Yeon Lee, Dasom Kim, Im Joo Rhyu, Dong Hwee Kim

**Affiliations:** a Department of Physical Medicine and Rehabilitation, College of Medicine, Korea University, Ansan, Republic of Korea; b Department of Internal Medicine, Division of Oncology/Hematology, Korea University Ansan Hospital, Korea University College of Medicine, Ansan, Republic of Korea; c Department of Anatomy, Korea University College of Medicine, Seoul, Republic of Korea; d Division of Brain Korea 21 Plus Program for Biomedical Science, Korea University College of Medicine, Seoul, Republic of Korea.

**Keywords:** anatomical landmarks, cadaver study, electromyography, flexor digitorum profundus, needle electrode, neurovascular structures

## Abstract

The flexor digitorum profundus (FDP) muscle plays a crucial role in finger flexion and is innervated by the anterior interosseous and ulnar nerves. Electromyographic evaluation of the FDP is essential in diagnosing neuromuscular disorders but carries a risk of neurovascular injury due to the complexity of accurate needle placement. This study aimed to evaluate the anatomical feasibility of a landmark-based needle insertion technique for the index- and middle-finger portions of the FDP in fresh cadaveric specimens by assessing targeting accuracy and the distances from the insertion point to the median nerve and ulnar artery. A cadaveric study was conducted using 21 upper limbs from 11 specimens. Needles were inserted into FDP 1 (digit 2) and FDP 2 (digit 3), using the palmaris longus tendon as a key anatomical landmark. India ink injections were performed at the needle tip to verify correct placement relative to neurovascular structures. The accuracy of placement, distances from the median nerve and ulnar artery, and muscle widths were measured. Anatomical targeting was successful for FDP 1 (digit 2) in 19 of 21 limbs (90.5%) and for FDP 2 (digit 3) in 17 of 21 limbs (81%). The median horizontal distances from the needle insertion point to the median nerve and ulnar artery were 7.0 mm and 9.5 mm, respectively, providing anatomical reference data for needle placement. The median muscle widths were 8.8 mm for FDP 1 and 9.4 mm for FDP 2. These findings support the feasibility of landmark-based needle placement in the FDP, with a high anatomical targeting rate for FDP 1. This anatomical landmark-based technique may provide a useful option for electromyographic evaluation of the FDP, particularly FDP 1, when real-time imaging is unavailable. However, FDP 2 showed lower targeting accuracy. Further clinical studies are needed to evaluate the safety and practicality of this technique in patients.

## 1. Introduction

The flexor digitorum profundus (FDP) muscle is crucial for finger movements and is innervated by both the anterior interosseous nerve (AIN) and the ulnar nerve. Its tendons pass through the carpal tunnel to attach to the distal phalanges of the second to fifth digits.^[1]^ For clarity, the tendons of FDP muscle are designated as follows: the portions inserting on the distal phalanges of digits 2 and 3 are referred to as FDP 1 and FDP 2, respectively, while those inserting on digits 4 and 5 are designated as FDP 3 and FDP 4. FDP 1 usually receives innervation from the AIN alone, whereas FDP 2 frequently receives dual innervation from the AIN and ulnar nerve. FDP 3 and FDP 4 most commonly receive innervation from the ulnar nerve alone.^[2]^ Although the flexor pollicis longus and pronator quadratus are important muscles in evaluating AIN dysfunction, selective involvement of the index-finger FDP has been reported.^[3]^ This provides a rationale for directly assessing FDP 1 when its involvement is suspected. However, incorrect needle placement during electromyographic studies can result in recordings from the ulnar portion of the muscle.

Several needle insertion techniques for targeting the radial portion of the FDP have been proposed. Standard (EMG) electromyography references describe approaches from the ulnar side of the forearm,^[4–7]^ but these methods lack clear guidance on the depth required to reach the desired radial portion, increasing the risk of recording from FDP 3 and 4. Burgar et al suggested inserting a needle into FDP 1 halfway between the elbow and wrist creases, advancing it through the flexor carpi radialis to a depth of approximately 30 mm.^[8]^ However, this technique carries risks of damaging the median nerve, located between the flexor digitorum superficialis and FDP, as well as the flexor pollicis longus, radial artery, and superficial radial nerve.

Ryou et al used ultrasonographic measurements to propose palmaris longus (PL) tendon-based needle trajectories for FDP 1 and FDP 2 at the junction of the middle and distal thirds of the volar forearm.^[9]^ On the ultrasound images, trajectories of 61.7° and 90° intersected FDP 1 and FDP 2 in 91.4% and 90.0% of forearms, respectively, without crossing the median nerve or ulnar artery. However, the study did not include actual needle insertion.

Building on the ultrasonographic measurements reported by Ryou et al^[9]^ the present study assessed the anatomical feasibility of applying the proposed landmark-based angles in fresh cadaveric specimens. We evaluated whether the needles reached the intended FDP portions and measured their anatomical relationships with nearby neurovascular structures. These findings provide anatomical data relevant to the potential use of the approach when real-time imaging is unavailable.

## 2. Methods

Twenty-one upper limbs from a convenience sample of 11 fresh cadavers (10 male and 1 female) were included in the study. The sample size was determined by the availability of cadaveric specimens. The cadavers were provided by the Department of Anatomy, Korea University College of Medicine, Seoul, Republic of Korea. Data collection was conducted in 2019, before the amendment to the Act on Dissection and Preservation of Corpses (Act No. 17203) took effect on April 8, 2021. Accordingly, Institutional Review Board review was not required for this cadaveric study. The specific site of interest was the junction of the middle and distal thirds of the volar forearm. Specimens were positioned supine with forearms supinated and elbows fully extended.

With the skin intact, the first needle was inserted immediately medial (ulnar) to the PL tendon, perpendicular to the skin (90°), to target FDP 2 (digit 3), and 0.1 cc of India ink was injected. After removing the first needle, a second needle was inserted at the same site and directed radially at 61.7° relative to the horizontal volar surface of the forearm in the transverse plane to target FDP 1 (digit 2) (Fig. 1B). These insertion angles were based on the study by Ryou et al^[9]^.

**Figure 1.**
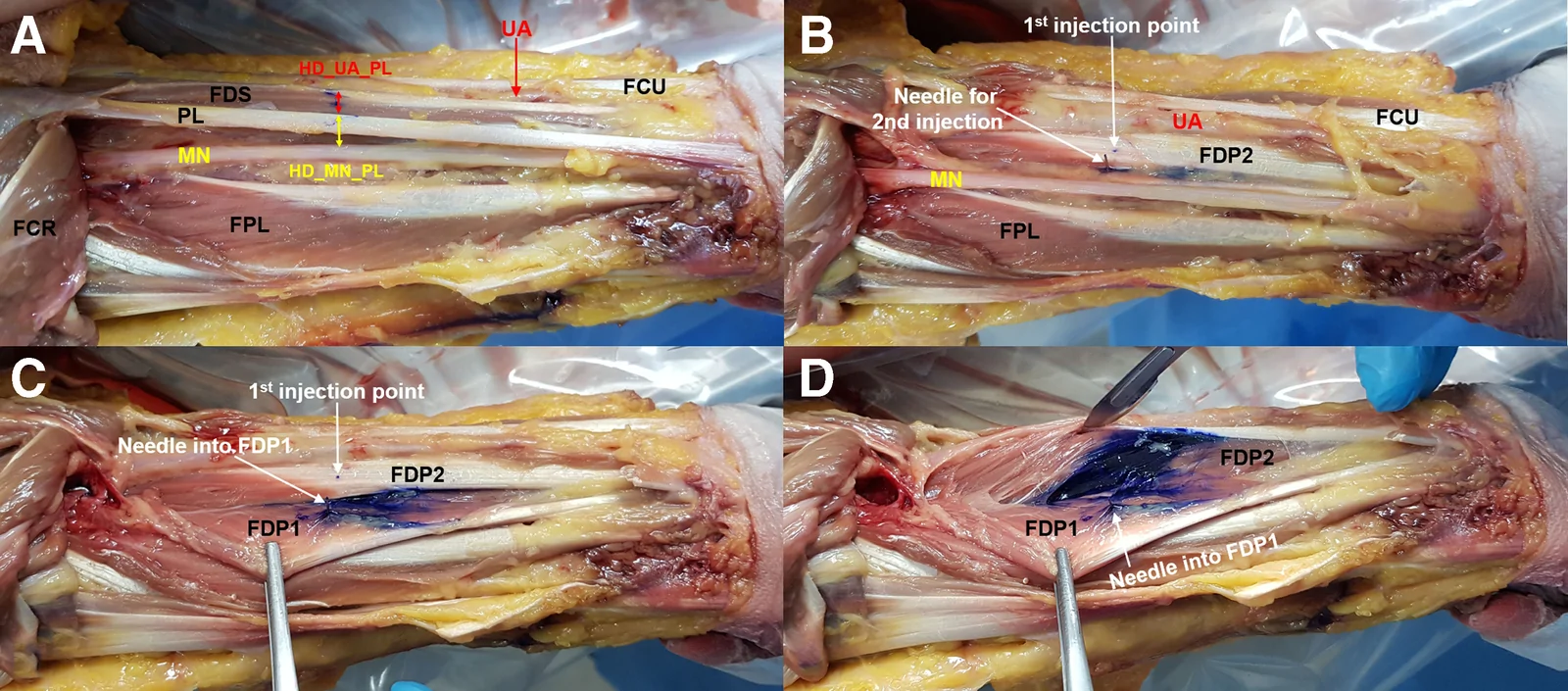
Anatomical relationship between the FDP and needle. The skin and flexor carpi radialis have been removed. The flexor digitorum superficialis, ulnar artery, flexor carpi ulnaris, palmaris longus, median nerve, and flexor pollicis longus are exposed. The horizontal distance of the needle to the median nerve (HD_MN_PL) and the horizontal distance of the needle to the ulnar artery (HD_UA_PL) are shown in panel (A) The first needle was inserted immediately medial (ulnar) to the palmaris longus tendon, perpendicular to the skin (90°), to target FDP 2. The second needle was inserted at the same site and directed radially at 61.7° relative to the horizontal volar surface of the forearm in the transverse plane to target FDP 1; 0.1 cc of India ink was injected through each needle (B). The number of cases in which the ink was on FDP 1 (C) or FDP 2 (D) was counted. FCR = flexor carpi radialis, FDS = flexor digitorum superficialis, FCU = flexor carpi ulnaris, FDP = flexor digitorum profundus, FPL, flexor pollicis longus, MN = median nerve, PL = palmaris longus, UA = ulnar artery.

After needle placement, the relationship between the needle, the median nerve, and FDP 1 was evaluated using ultrasonography, without repositioning the needle. The skin and flexor carpi radialis muscle were then removed to expose the flexor digitorum superficialis, ulnar artery, flexor carpi ulnaris, PL, median nerve, and flexor pollicis longus for anatomical examination (Fig. 1A).

Horizontal distances from the needle insertion point to the median nerve (HD_MN_PL) and ulnar artery horizontal distance from the needle insertion point to the ulnar artery were measured to characterize the anatomical relationships between the insertion point and these neurovascular structures. Successful needle entry into the FDP 1 muscle was recorded (Fig. 1C), as was the marking of the FDP 2 muscle with India ink (Fig. 1D). Additionally, the widths of FDP 1 (Width_FDP1) and FDP 2 (Width_FDP2) muscles were measured to evaluate anatomical variability and targeting precision.

### 2.1. Statistical analysis

Descriptive statistics were employed to summarize the anatomical and procedural findings. Continuous variables, such as forearm length, horizontal distances from the needle insertion point to the median nerve and ulnar artery, and the widths of FDP 1 and FDP 2 muscles, were expressed as medians with ranges (minimum–maximum) to account for variability in the sample. The success rates for needle placement into FDP 1 and FDP 2 were calculated as percentages, representing the proportion of correctly targeted injections relative to the total attempts. Data analysis was performed using MedCalc statistical software (MedCalc Software Ltd., Ostend, Belgium).

## 3. Results

The median forearm length in this study was 24.5 cm, with a range of 22.0 to 27.0 cm. The median horizontal distance from the needle insertion point to the median nerve was 7.0 mm (range: 1.6–11.4 mm), while the distance to the ulnar artery was 9.5 mm (range: 5.8–24.2 mm). The median widths of the FDP 1 and FDP 2 muscles were 8.8 mm (range: 5.7–14.6 mm) and 9.4 mm (range: 5.4–14.0 mm), respectively, as shown in Table 1.

**Table 1:** Anatomical Relationships between Flexor Digitorum Profundus (Digits 2 and 3) and neurovascular structures.

	Median	Minimum	Maximum
Forearm length (cm)	24.5	22.0	27.00
HD_MN_PL (mm)	7.0	1.6	11.4
HD_UA_PL (mm)	9.5	5.8	24.2
Width_FDP1 (mm)	8.8	5.7	14.6
Width_FDP2 (mm)	9.4	5.4	14.0

HD_MN_PL = horizontal distance between the median nerve and ulnar side of the palmaris longus (PL) tendon; HD_UA_PL = horizontal distance between the ulnar artery and ulnar side of the PL tendon; Width_FDP1 = width of flexor digitorum profundus 1, Width_FDP2 = width of flexor digitorum profundus 2.

Anatomical targeting was successful for FDP 1 in 19 of 21 limbs (90.5%) and for FDP 2 in 17 of 21 limbs (81%) (Fig. 2).

**Figure 2.**
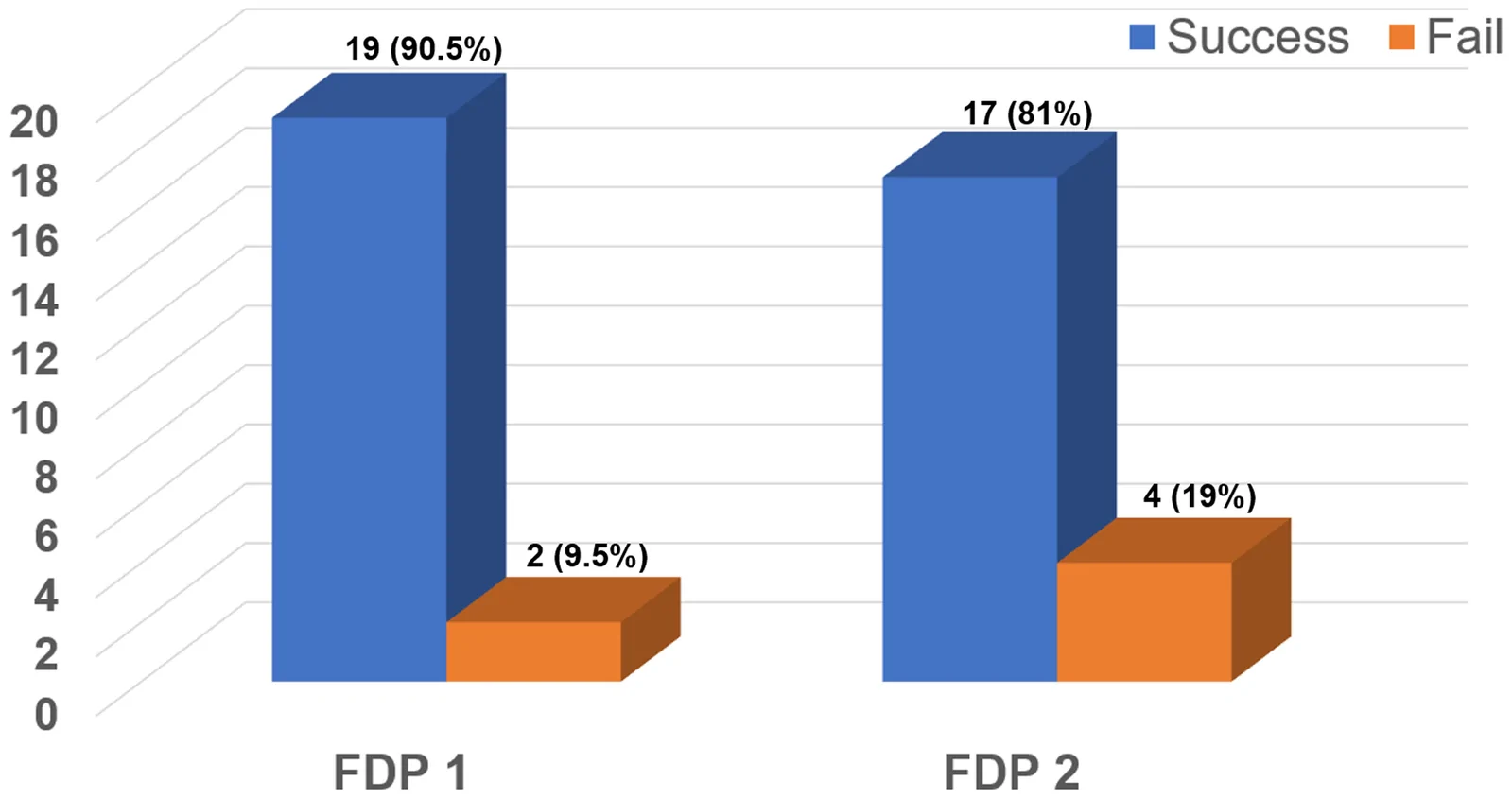
Comparison of success and failure rates in 2 groups: FDP 1 and FDP 2. For FDP 1, 19 cases (90.5%) were successful, while 2 cases (9.5%) failed. For FDP 2, 17 cases (81%) were successful, whereas 4 cases (19%) failed. The blue bars represent successful outcomes, and the orange bars represent failures, with percentages labeled for each group. FDP = flexor digitorum profundus, FPL = flexor pollicis longus.

## 4. Discussion

The median horizontal distances from the needle insertion point to the median nerve and ulnar artery were 7.0 mm and 9.5 mm, respectively, with minimum distances of 1.6 mm and 5.8 mm. The minimum horizontal distance of 1.6 mm underscores the need for caution because of the proximity of the median nerve to the insertion point. In clinical practice, slow, controlled needle advancement with attention to patient-reported symptoms is advisable as a procedural precaution.^[10]^ However, the present cadaveric study did not evaluate whether these measures reduce nerve injury risk.

Anatomical targeting of FDP 1 was achieved in 19 of 21 limbs (90.5%) (Fig. 2). The median muscle widths of FDP 1 and FDP 2 were 8.8 mm and 9.4 mm, respectively, describing the target dimensions at the selected forearm level. Together, these anatomical findings support the feasibility of using the PL tendon as a landmark for FDP needle placement.

Traditional anterior and medial insertion approaches to the FDP involve different depths and anatomical considerations, as described by Heales et al^[11]^. The present PL tendon-based approach achieved anatomical targeting of FDP 1 in 19 of 21 limbs (90.5%), supporting its potential as a landmark-based option. However, this study did not directly compare the approaches or evaluate neurovascular injury.

Previous studies reported lower FDP placement proportions: 66.7% (75.0% after adjusted classification) in an embalmed cadaveric study by Goodmurphy et al and 65.9% for initial manual placement in a clinical study by Picelli et al ^[12,13]^. These proportions are numerically lower than the 81% observed for FDP 2 in the present study. However, differences in study design and target definition limit direct comparison.

Anatomical variability within the FDP muscle further highlights the importance of precise targeting. Hwang et al reported that FDP 1 is predominantly innervated by the AIN, whereas FDP 2 is more likely to receive dual innervation.^[2]^ Additionally, Hodde et al identified areas of overlapping activity between FDP 2 and FDP 3 using ultrasound, demonstrating the complexity of innervation patterns within the muscle.^[14]^ Reilly and Schieber’s findings that FDP 1 is independently activated during finger flexion further underscore the clinical relevance of accurately isolating FDP 1 for electromyographic studies.^[15]^ These findings collectively suggest that FDP 1 is a key muscle for diagnosing AIN neuropathy and localizing radial portion dysfunctions.

The anatomical targeting results may have clinical relevance for the electrodiagnostic evaluation of AIN neuropathy because selective involvement of the index-finger FDP has been reported even when flexor pollicis longus and pronator quadratus findings were normal.^[3]^ The observed 90.5% targeting rate for FDP 1 suggests that the PL tendon-based approach may be useful when real-time imaging is unavailable. Further clinical studies incorporating actual needle EMG recordings are needed to evaluate its diagnostic performance and procedural safety.

### 4.1. Study limitations

While the findings of this study are promising, several limitations warrant consideration. The use of fresh cadaveric specimens is a strength because it allowed needle placement to be assessed in tissue conditions that more closely approximate clinical practice than embalmed specimens. The anatomical targeting results may have practical value when real-time imaging is unavailable.

First, the PL tendon, which serves as the key anatomical landmark in this technique, is absent in approximately 25% of individuals. This anatomical variation highlights the importance of confirming the presence of the PL tendon prior to the procedure. In cases where the PL tendon is absent or poorly defined, alternative landmarks or techniques may need to be considered.

Second, this study was conducted on a limited sample size of cadaveric specimens under controlled conditions. The limited sample restricts the precision of the targeting proportions and the generalizability of the observed anatomical relationships. Cadaveric specimens lack the dynamic characteristics of live tissue, such as vascular pulsation, muscle contraction, and tissue compliance, which may influence needle placement and safety during clinical application. Furthermore, the specimens used did not exhibit pathological conditions, such as scarring, systemic diseases, or previous injuries, which could affect the generalizability of the findings.

Third, FDP 2 was successfully targeted in 17 of 21 limbs (81%), with 4 unsuccessful placements (19%). The 4 unsuccessful placements remain a limitation of selective middle-finger FDP targeting and should be considered when interpreting the potential clinical application of the approach.

Finally, needle placement was confirmed anatomically using India ink, and EMG recordings were not performed. Therefore, the present findings demonstrate anatomical targeting but do not establish functional EMG performance or diagnostic reliability. These outcomes should be evaluated in future clinical studies.

## 5. Conclusion

This cadaveric study supports the anatomical feasibility of a PL tendon-based approach at the junction of the middle and distal thirds of the volar forearm for targeting the FDP 1 and 2 muscles. In this study, insertion angles of 61.7° and 90° were used to target FDP 1 and FDP 2, respectively. Successful anatomical targeting of FDP 1 in 19 of 21 limbs (90.5%) supports the 61.7° approach as a potential option for accessing the index-finger portion of the FDP. Clinical safety and EMG performance require further validation. However, in cases where the PL tendon is absent, a conventional proximal approach may be considered.

## Author contributions

**Data curation:** Hong Bum Park MD, Se Ryeon Lee, Jun Yeon Lee, Dasom Kim, Dong Hwee Kim.

**Methodology:** Hong Bum Park MD, Jun Yeon Lee, Dasom Kim.

**Supervision:** Im Joo Rhyu, Dong Hwee Kim.

**Conceptualization:** Dong Hwee Kim.

**Writing – original draft:** Se Ryeon Lee.

**Writing – review & editing:** Im Joo Rhyu, Dong Hwee Kim.
